# Marfan syndrome and cardiovascular complications: results of a family investigation

**DOI:** 10.1186/s12872-017-0629-8

**Published:** 2017-07-19

**Authors:** Simon Antoine Sarr, Siddikatou Djibrilla, Fatou Aw, Malick Bodian, Kana Babaka, Aliou Alassane Ngaidé, Momar Dioum, Serigne Abdou Ba

**Affiliations:** 1 Department of Cardiology, Teaching Hospital Aristide Le Dantec, PO Box 6003, Dakar Etoile, Sénégal; 2 Department of Cardiology, General Hospital of Grand Yoff, Dakar, Sénégal; 3 Department of Cardiology, Fann Teaching Hospital, Dakar, Sénégal

## Abstract

**Background:**

Cardiovascular complications in Marfan syndrome (MFS) make all its seriousness. Taking as a basis the Ghent criteria, we conducted a family screening from an index case. The objective was to describe the clinical characteristics of MFS anomalies and to detect cardiovascular complications in our patients.

**Case presentation:**

Six subjects were evaluated. Patients had to be in the same uterine siblings of the index case or be a descendant. The objective was to search for MFS based on the diagnostic criteria of Ghent and, subsequently, detecting cardiovascular damage. The average age was 24 years. The examination revealed three cases of sudden death in a context of chest pain. Five subjects had systemic involvement with a score ≥ 7 that allowed to the diagnosis of MFS. Two patients had simultaneously ectopia lentis and myopia. In terms of cardiovascular damage, there were three cases of dilatation of the aortic root, two cases of aortic dissection of Stanford’s type A with severe aortic regurgitation in one case and moderate in the other. There were three patients with moderate mitral regurgitation with a case by valve prolapse.

**Conclusion:**

The family screening is crucial in Marfan syndrome. It revealed serious cardiovascular complications including sudden death and aortic dissection.

## Background

Marfan syndrome (MFS) is a genetic disease with autosomal dominant transmission, usually related to a mutation in the fibrillin gene type 1. The possibility of cardiovascular complications justifies a systematic family screening when a case is discovered [1]. This syndrome is characterized by musculoskeletal, cardiovascular and ocular damages. Its diagnosis is based on the Ghent criteria [2] which are of great importance in a context where the genetic study is inaccessible, expensive with a long waiting time of results.

Taking as a base the new Ghent criteria [2], we conducted a family screening from an index patient in whom the diagnosis of MFS was made. The objectives were to look for clinical anomalies characteristics of MFS and detect cardiovascular complications related, in the family.

### Case presentation

We were interested in relatives of a patient with MFS and cardiovascular damage, at the Teaching Hospital Aristide Le Dantec in Dakar, from January to March 2015. Patients had to be in the same uterine siblings of the index case. Their descendants were also included. Relatives who refused to participate in the study were not included. We identified the siblings of the index case and had made a briefing on the MFS and its complications. The objectives of the work were specified and the consent of the subjects required. Subsequently, subjects were examined clinically and complementary explorations carried out. Computed tomography was made in a case of anomaly indicating namely the dilation of an aortic segment or the existence of an aortic dissection.

### Ethical considerations

This study showed no risk for participants, its purpose was clear to them so they can give their consent. A sheet was prepared for this. The results were communicated to them and support was offered.

### Parameters studied

- The clinical evaluation focused on:
- Search of functional signs,
- The following constants and anthropometric data: weight, height, body mass index, waist circumference, blood pressure in both arms, the arm span on height ratio, the upper segment on the lower segment (US / LS).
- A complete physical examination, which was particularly interested in cardiovascular, morpho-skeletal, and ocular abnormalities in search of new diagnostic criteria of Ghent.

The diagnosis was selected according to the following different possibilities [3]:

In the absence of family history.

1. Aortic root dilatation (Z score ≥ 2) or aortic dissection and ectopia Lentis = Marfan syndrome.

2. Aortic root dilatation (Z score ≥ 2) or aortic dissection and FBN1 = Marfan syndrome.

3. Aortic root dilatation (Z score ≥ 2) or aortic dissection and systemic score ≥ 7 points = Marfan syndrome.

4. Ectopia lentis and FBN1 with known aortic root dilatation or aortic dissection = Marfan syndrome.

In the presence of family history.

5. Ectopia lentis and family history of Marfan syndrome (as defined above) = Marfan syndrome.

6. A systemic score ≥ 7 points and family history of Marfan syndrome = Marfan syndrome.

7. Aortic Root Dilatation (Z score ≥ 2 above 20 years old, ≥ 3 below 20 years old) or aortic dissection + family history of Marfan syndrome = Marfan syndrome.

All subjects had been consulted by an ophthalmologist (research lens dislocation, myopia ...)

- The paraclinical evaluation consisted of achieving:
  - A resting electrocardiogram (ECG)

The analysis clarified the nature of rhythm, looking for abnormal rhythm or conduction disturbances of repolarization or signs of atrial or ventricular hypertrophy.

- A transthoracic echocardiography

The review was done by a single operator who was looking for dilation, with or without aortic dissection. The measurements were carried out successively in longitudinal section major axis (aortic root), in the supra-sternal cut (butt) and sectional subcostal (abdominal aorta). We also were interested in valvular structures in search of ballooning or prolapse, valvular leakage. Cavitary dimensions were also appreciated as the systolic function of both ventricles.

- Radiological examinations

- The chest X-ray was looking for pneumothorax.

- The pelvis X-ray was looking for acetabular protrusion.

- The chest computed-tomography was done in a case of abnormalities of the aorta Doppler echocardiography. It specified dimensions of the aorta, the existence and extent of aortic dissection as well as its complications.

After the collection of clinical and laboratory data, we calculated the systemic involvement score (Table 1).

**Table 1:** Systemic score of Marfan syndrom

Systemic involvement	Points
Wrist and thumb sign	3
Wrist or thumb sign	1
Pectus carinatum deformity	2
Pectus excavatum or chest asymmetry	1
Hindfoot deformity	2
Plain flat foot	1
Spontaneous pneumothorax	2
Dural ectasia	2
Protusio acetabuli	2
Scoliosis or thoracolumbar kyphosis	1
Reduced elbow extension	1
3 of 5 facial features	1
Skin striae	1
Severe myopia (> 3 diopters)	1
Mitral valve prolapse	1
Reduced upper segment / lower segment (US/LS) and increased arm span / height	1

(Facial features: dolicocephaly, malar hypoplasia, enophthalmos, retrognathia, and down-slanting palpebral fissures)

Maximum total: 20 points; a score ≥ 7 is considered a positive systemic score

After clinical evaluation and paraclinical, according to found damages, they were offered medical care and / or surgical.

### Epidemiological aspects

In total, six subjects were screened. All were male. The average age was 24 years, with extremes of 14 and 42 years. We reported three cases of sudden death in the family: mother and two half-brothers of the index case. Among these, the deaths had occurred in a context of physical effort and preceded by chest pain. One of the subjects was an active smoker of 6 packs per year. They were of low socioeconomic status and had no support. Three of them were students, two were fishermen and one was a gardener.

### Clinical aspects

The size was an average of 179 cm with extremes of 168 and 192 cm.

Two patients (2/6) had functional signs: one had palpitations, and the other had episodic precordialgia not related to effort.

Two patients had a heart murmur. It was in one, a diastolic murmur latero-sternal 3/6, and, in the other, an apical systolic murmur 2/6.

**Fig. 1:**
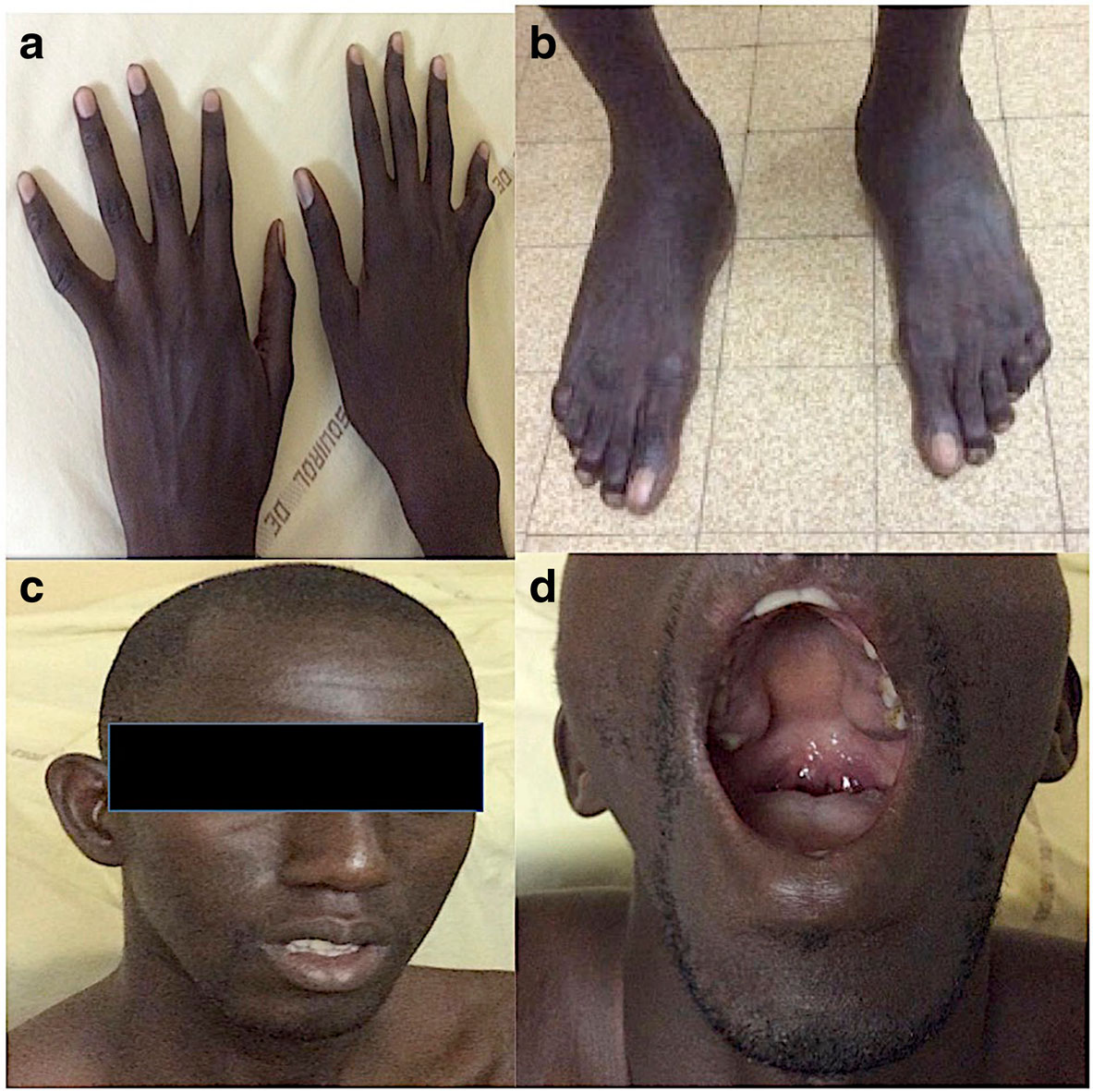
Clinical anomalies of a patient. **a** arachnodactyly; **b**: plain flat foot (**c**) dolicocephaly and malar hypoplasia; **d** High arched palate

Five patients (5/6) had systemic involvement with a score ≥ 7 (Fig. 1). The ocular examination showed ectopia lentis and coexisting myopia in two patients (2/6) (Table 2).

**Table 2:** Summary of clinical features of the patients

Parameters	P1	P2	P3	P4	P5	P6	Total
Height	1.88	1.83	1.78	1.92	1.68	1.69	Mean 1.79
Arm span/height ratio > 1, 05	yes	yes	yes	yes	no	no	4
Reduced US/LS	yes	yes	yes	yes	yes	no	5
Face:							
- Dolicocephaly	yes	yes	yes	yes	yes	no	5
- Enophtalmos	yes	yes	yes	yes	yes	no	5
- Down-slanting palpebral fissures	yes	no	no	no	yes	no	1
- Malar hypoplasia	yes	yes	yes	yes	yes	no	5
- Retrognathia	yes	yes	yes	yes	yes	no	5
Pectus carinatum	no	no	no	no	no	no	0
Pectus excavatum	yes	yes	yes	no	yes	no	4
Thumb sign	yes	yes	no	yes	yes	no	4
Wrist thumb	yes	yes	no	no	yes	no	3
Scoliosis	no	no	no	no	no	no	0
Thoracolumbar kyphosis	no	no	no	yes	yes	no	2
Plain plat foots	yes	yes	yes	yes	yes	no	5
Reduced elbow extension	yes	no	yes	no	no	no	2
Skin striae	yes	yes	yes	yes	yes	no	5

*P* patient

*US*/*LS* upper segment/lower segment ratio

### Paraclinical aspects

**Fig. 2:**
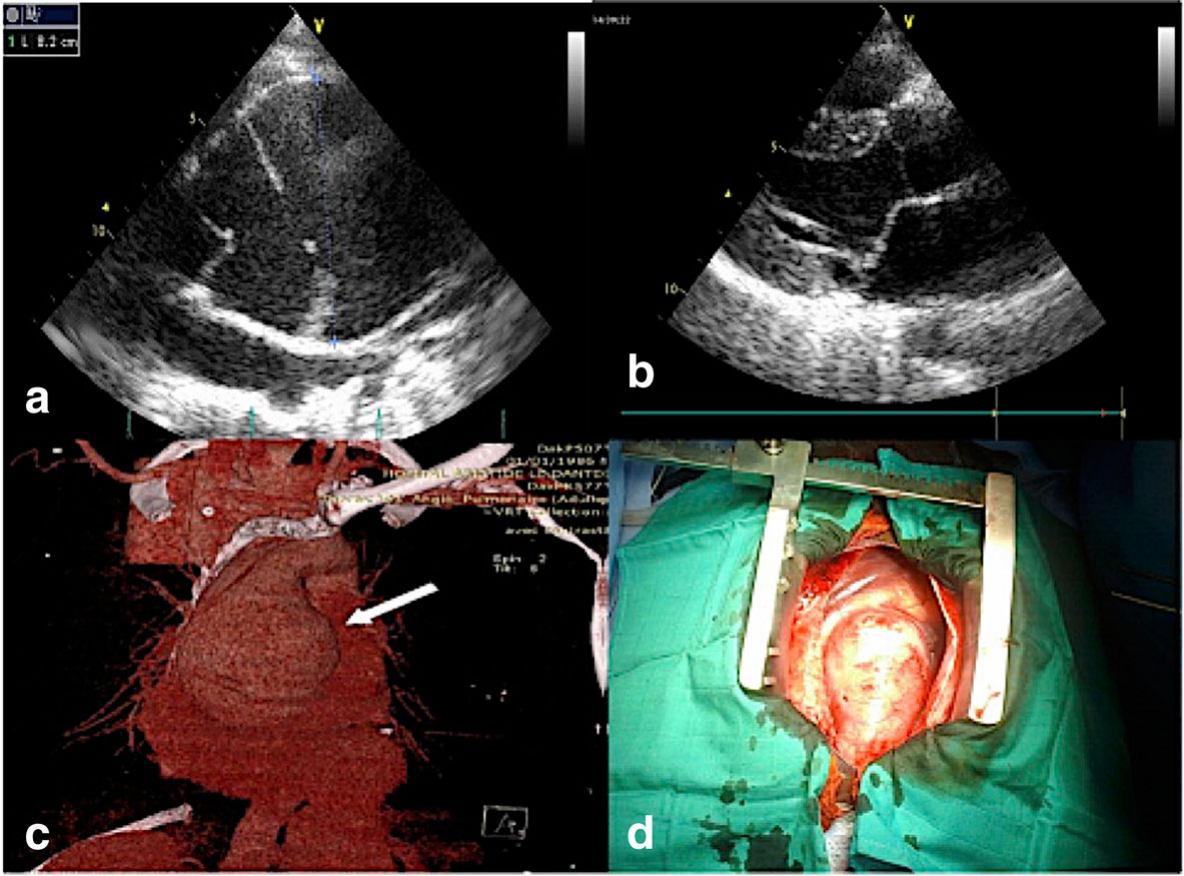
Images of aortic dissection of a patient. **a** Transthoracic echocardiography parasternal long axis view showing the dilation of aorta and the intimal flap; **b** Transthoracic echocardography parasternal long axis view showing a mitral valve prolapse; **c** Computed-tomography scan showing an aortic dissection; **d** Cardiac surgery of the patient

The echocardiography highlighted dilation of the aortic root in two cases. There were two cases of aortic dissection of Stanford’s type A with severe aortic regurgitation in one and moderate in the other (Fig. 2). There were also three cases of moderate mitral regurgitation with a case of mitral valve prolapse. The aortic abnormalities were confirmed by computed tomography. Various anomalies are summarized in Table 3. The application of the Ghent criteria supported the findings of MFS in 5 of 6 cases as shown in Table 4.

**Table 3:** Summary of paraclinical features

Anomalies	P1 (index case)	P2	P3	P4	P5	P 6	Number
Left ventricular hypertrophia	yes	no	no	no	no	no	1
Left ventricular dilatation	yes	no	no	no	no	no	1
Aortic dissection	yes	no	no	yes	no	no	2
Aortic dilatation	yes	yes	no	yes	no	no	3
Aortic regurgitation	yes	no	no	yes	no	no	2
Mitral valve prolapse	no	no	no	no	yes	no	1
Mitral regurgitation	no	yes	yes	no	yes	no	3
Protrusio acetabulae	no	yes	no	yes	no	no	2

*P* patient

**Table 4:** Ghent criteria for our patients

		P1 (index case)	P2	P3	P4	P5	P6
Ascending aorta	Dilatation	yes: Z score 3,01	yes	no	yes	no	no
	Dissection	yes	no	no	yes	no	no
Ectopia lentis		yes	no	yes	no	no	no
Systemic involvement		yes	yes	yes	yes	yes	no
Family history		no	yes	yes	yes	yes	yes
Diagnosis of Marfan syndrom retained		yes	yes	yes	yes	yes	no

*P* patients

### Therapeutic aspects

All patients in whom the diagnosis of MFS was retained ha a beta blocker. A subject (1 subject) had a surgical repair of aortic dissection by Bentall procedure with a good result in more than a year now. He is, moreover, under effective anticoagulation.

The second case of aortic dissection (about 4) could not be operated until now for lack of funds. Annual monitoring clinical and paraclinical (aortic root, changes in valvular heart disease).

Subjects 1 and 3 are awaiting surgical treatment of the eye.

## Discussion and conclusion

MFS is characterized by various musculoskeletal violations. They are at the forefront of clinical expression.

These include, among other things, the dolichostenomelia using the arm span on height ratio. This ratio normally equal to 1, is around 1.03 in 80% of patients and becomes a criterion from 1.05 [3]. In our work, this ratio was increased in 4/6 subjects. Signs of the thumb and wrist are the witnesses of the arachnodactyly as well as ligamentous laxity. [3] Regarding chest deformities, 2 subjects had kyphosis (subjects 4 and 5). Subjects with MFS have valgus flat feet since childhood [3, 4], a result of laxity. This sign was standing in the subjects studied.

Acetabular protrusion was found in 2 subjects. Kwang find them in 77% of reported cases. However, its presence does not have an impact on the final diagnosis because of its etiology which is variable and does not correlate to the presence of ectopia lentis or aortic disease [5]. Cardiovascular damage will determine the vital prognosis due to the risk of aortic dissection, often preceded by dilation and valvular disease [6]. They were described in 1943 by Bear, Tausig and Oppenheimer who reported two cases of sudden death in young adults with two dolichostenomelia and fusiform aneurysm of the ascending aorta. The histological examination had revealed the causal lesion of cardiovascular events like medial necrosis described by Gsell and Erdhein [7].

Acute aortic dissection is the most dangerous complication and the most common cause of death. The risk of dissection of the ascending aorta increases with the degree of aortic dilatation. The dilatation of the ascending aorta is observed in 60–80% of patients with MFS. It also represents a major diagnostic criterion. Conventionally it interests the sinus of Valsalva realizing expansion “in onion bulb” and the proximal portion of the ascending aorta [8]. This dilatation can affect the descending aorta in a minority of patients [9].

Aortic dissection usually occurs in the ascending aorta, but can extend at the butt of the neck vessels and the descending aorta [10].

It is more likely to occur if:

- The dilatation is important: it is considered that the risk is low (though not zero) when the aortic diameter at the sinuses of Valsalva remains below 50 mm. Similarly, it is outstanding in the absence of dilation. The aortic diameter is regarded as the most powerful predictor of aortic dissection [11];

- The dilatation is fast: the examination has to be repeated to confirm the value by another imaging technique;

- Aortic dilatation is diffuse and extends beyond the sino-tubular junction.

- A parent who experienced aortic dissection without significant dilatation;

- The existence of high blood pressure.

The dissection of the descending aorta without ascending aortic dissection is rare because it is usually an extension of the ascending dissection [9].

This damage of the aorta exposes to his break and thus to sudden death. This complication is one of the assumptions in cases of sudden death recorded (2 cases).

A valve disease can also be observed in the MFS. The aortic type may be related to the dilation of the aortic root in the absence of valvular structural abnormality. There is, in fact, a misalignment of the semilunar aortic valve by the deformation of the aortic root. Aortic regurgitation can also complicate a proximal aortic dissection [12].

The mitral involvement is common but is generally limited to prolapse with minimal or moderate leakage. It was noted in three cases in our work. There was a case of valve prolapse.

Moreover, it seems that the prevalence of ventricular and supraventricular arrhythmias was higher in patients with MFS than in the general population, even in the absence of valvular leakage [13].

Rare cases of cardiac dilatation unrelated to a possible regurgitation were reported as a few cases of conduction disturbances. They might reflect a primary abnormality of the heart muscle [6, 14].

The indication for surgery was put in 2 patients for aortic dissection of Stanford’s type A (subjects 1 and 4). Only about 1 had a Bentall procedure, this after a fundraiser. This is an expensive procedure, therefore, inaccessible to our subjects of low socioeconomic level.

The surgery was life-saving for patients with MFS. Indeed, the average life expectancy which was 32 years is now close to that of the general population due to early surgery [15].

This prognostic improvement is the fact of several parameters. These include a better understanding of the disease, family screening but also the most accurate assessment of aortic risk authorized by imaging techniques such as transthoracic echocardiography and cardiac magnetic resonance imaging [15].

The family screening is crucial in Marfan syndrom. Diagnostic criteria have helped to detect the disease in 5 patients of 6. Similarly, it has revealed serious cardiovascular complications including sudden death and aortic dissection.

## Abbreviation

MFS: Marfan syndrome
